# Exploiting labels from multiple experts in automated sleep scoring

**DOI:** 10.1093/sleep/zsad034

**Published:** 2023-02-16

**Authors:** Samaneh Nasiri, Wolfgang Ganglberger, Haoqi Sun, Robert J Thomas, M Brandon Westover

**Affiliations:** Department of Neurology, Massachusetts General Hospital (MGH), Boston, MA, USA; Harvard Medical School, Boston, MA, USA; Clinical Data Animation Center (CDAC), Boston, MA, USA; McCance Center for Brain Health, MGH, Boston, MA, USA; Harvard Medical School, Boston, MA, USA; Clinical Data Animation Center (CDAC), Boston, MA, USA; McCance Center for Brain Health, MGH, Boston, MA, USA; Harvard Medical School, Boston, MA, USA; Clinical Data Animation Center (CDAC), Boston, MA, USA; McCance Center for Brain Health, MGH, Boston, MA, USA; Department of Medicine, Division of Pulmonary, Critical Care & Sleep, Beth Israel Deaconess Medical Center, Boston, MA, USA; Harvard Medical School, Boston, MA, USA; McCance Center for Brain Health, MGH, Boston, MA, USA; Department of Neurology, Massachusetts General Hospital (MGH), Boston, MA, USA; Harvard Medical School, Boston, MA, USA; Clinical Data Animation Center (CDAC), Boston, MA, USA; McCance Center for Brain Health, MGH, Boston, MA, USA; Department of Medicine, Division of Pulmonary, Critical Care & Sleep, Beth Israel Deaconess Medical Center, Boston, MA, USA

The current “ground truth” for sleep staging is manual scoring of the electroencephalogram following American Academy of Sleep Medicine (AASM) rules [1]. These rules specify how to label each 30-s epoch into one of five stages: Wake (W), Rapid Eye Movement (REM), and Non-REM 1–3 (N1, N2, and N3). However, AASM rules are not precise enough to be directly programmed into a computer. Moreover, NREM sleep from a biological standpoint exists along a continuous spectrum rather than in discrete stages [2]. This imprecision and artificial discretization lead to variable and imperfect inter-rater scoring agreement, ranging from 60% internationally to ~80% within the same institute [3]. Recently, several papers have developed deep neural networks for automated sleep staging [4–6]. This “AI-enabled sleep staging,” although proposed as a way to achieve objective and repeatable sleep staging, is ultimately limited by imprecision in the “gold standard” training labels. This is particularly true for AI methods which consider datasets annotated by a single scorer [7, 8]. One way to overcome the problem of noisy labels is to utilize datasets scored by multiple experts.

Most prior efforts to train sleep staging models using labels from multiple experts have combined labels using a simple majority vote scheme, which does not make optimal use of information about disagreement in voting among experts [9, 10]. In the current issue of *SLEEP*, Fiorillo et al. propose a framework for training deep learning algorithms that leverages labels from multiple experts more effectively than majority voting.

The authors adopt “label smoothing” to leverage multiple labels from different scorers efficiently [11]. Label smoothing assigns a non-zero probability to multiple classes, treating them as “soft,” as opposed to a baseline approach that uses “hard” labels, in which one class is treated as correct with 100% confidence [12]. In the baseline approach, hard labels are assigned by majority vote. In case of ties, the correct answer is taken to be the vote of the “most reliable” rater (the rater whose answers most frequently agrees with the majority). The paper then compares two label smoothing approaches to the baseline approach.

(1) *Label smoothing by a uniform distribution*: In this approach, if the majority label for a given epoch is wake (W), then the “hard label” would be $L=[p_{w},\;p_{N1},p_{N2},\;p_{N3},\;p_{REM}\;]\;=[1,0,0,0,0]$, where the 5 positions represent the probability that we assign to each of the 5 possible sleep stages. The smoothed label based on the uniform distribution would then be a mixture of the original hard label *L* and the uniform distribution vector $U=\left[\frac{1}{5},\frac{1}{5},\frac{1}{5},\frac{1}{5},\frac{1}{5}\right]$ where each stage is assigned a 1/5 probability, $L_{su}=\alpha L+(1-\alpha )U$. Here, the “mixing” parameter *α* is a number between 0 and 1 that determines how much weight is given to the hard label vector vs. the uniform distribution. This number is determined empirically. For illustration suppose this number *α* = 0.9, meaning that 90% of the weight is given to the hard label (majority vote) *L*, and 10% to the uniform distribution *U*. In this case, the smoothed label would be $L_{su}=[\begin{array}{lllll} 0.92 & 0.02 & 0.02 & 0.02 & 0.02 \end{array}\;]$. Note that most of the weight is still given to the label that received the majority vote, but the smoothed label allows for some uncertainty and thus might be expected to prevent the model from becoming overconfidence about the correct label for this example. Specifically, this smoothed label gives 90% of the total probability to the majority label and distributes the remaining 10% equally among the other possibilities. Note that the total weight (probability) of the smoothed label still adds up to one.
(2) *Label smoothing by soft consensus*: The second label smoothing strategy is based on Soft Consensus (SC). The SC for a given epoch is the vector containing the proportions of experts who voted for each stage. For example, if 6 experts labeled a given epoch, where 4 labeled the epoch as wake (W), and 2 as N1, then the SC vector would be $SC=\left[\frac{4}{6},\frac{2}{6},0,0,0\right]$. The smoothed label in this case would then be $L_{sc}=\alpha L+(1-\alpha )SC$. If we again assume as the value for the smoothing parameter *α* = 0.9, then the smoothed label is $L_{sc}=[\begin{array}{lllll} 0.97 & 0.03 & 0 & 0 & 0 \end{array}]$_._ This smoothed label again gives 90% of the probability to the majority label, but then distributes the remaining 10% unequally, according to the “soft consensus.”

For comparing the two label smoothing methods with the baseline majority voting approach, three open-access datasets scored by multiple physicians have been used; ISRC (Inter-scorer Reliability Cohort; *N* = 70 PSGs, *n* = 6 scorers) [13]; DOD-H (Dreem Open Dataset—Healthy; *N* = 25 PSGs, *n* = 5 scorers), and DOD-O (Dreem Open Dataset—Obstructive; *N* = 55 PSGs, *n* = 5 scorers) [10]. The authors used two deep learning-based sleep staging algorithms, DeepSleepNet-Lite (DSN-L) [14] and SimpleSleepNet (SSN) [10] to classify sleep stages into the five AASM sleep stages (Wake, REM, N1, N2, and N3). The authors used *K*-fold cross-validation for training each model (for ISRC, DOD-H, and DOD-0, *K* = 10, 25, and 10, respectively). During *K*-fold cross-validation, each dataset is split into *K* number of folds, onefold is considered as a test set, and the model is trained and validated on the remaining subjects’ data in *K* − 1 folds. This process is repeated until each fold takes a turn being the test set [15].

The authors use an averaged cosine similarity metric (ACS) to quantify the similarity between the hypnodensity graph generated by the models using label smoothing with SC and the hypnodensity graph generated by the scorer consensus (majority vote). The hypnodensity graph provides a probability distribution over sleep stages per epochs (i.e. each 30-s window). The authors used ACS to quantitatively evaluate the ability of the model to adapting to the consensus of the group of scorers, where a higher ACS value means a higher similarity between these two hypnodensity graphs. Based on ACS, the label smoothing by SC enabled both deep learning models to learn to perform substantially better than when label smoothing was not utilized, and better than label smoothing based on the uniform distribution.

A key limitation of this study is that the datasets used for training and evaluating the proposed method are small (*N* = 70, 25, 55 for three different datasets). To train a staging model that generalizes across clinically relevant parameters (e.g. age, gender, ethnicity, medical and neurological disorders) would require large datasets scored by multiple experts. However, this is challenging because no currently available datasets are large both in terms of number of patients and number of scorers. In this direction, crowdsourcing could be a viable solution to create larger multiply scored datasets [16]. Another limitation is that the number of experts needed to overcome the noise inherent in the human sleep staging process is not known. Finally, it is not clear how best to select a group of experts, although some guidance is available from other fields where crowd sourcing has proven effective; for example, the “crowd” should be large and diverse, and the judgments must be independent (e.g. from different institutions).

Despite these limitations, the proposed method is a welcome addition to the literature. Label smoothing provides a principled approach to leveraging the variability among multiple scorers to improve the performance of automated sleep scoring algorithms.
